# Supplementary figures and images for: SynBa: improved estimation of drug combination synergies with uncertainty quantification
Source: Bioinformatics. 2023 Jun 30;39(Suppl 1):i121–30. doi: 10.1093/bioinformatics/btad240 (PMC10311304; doi:10.1093/bioinformatics/btad240)

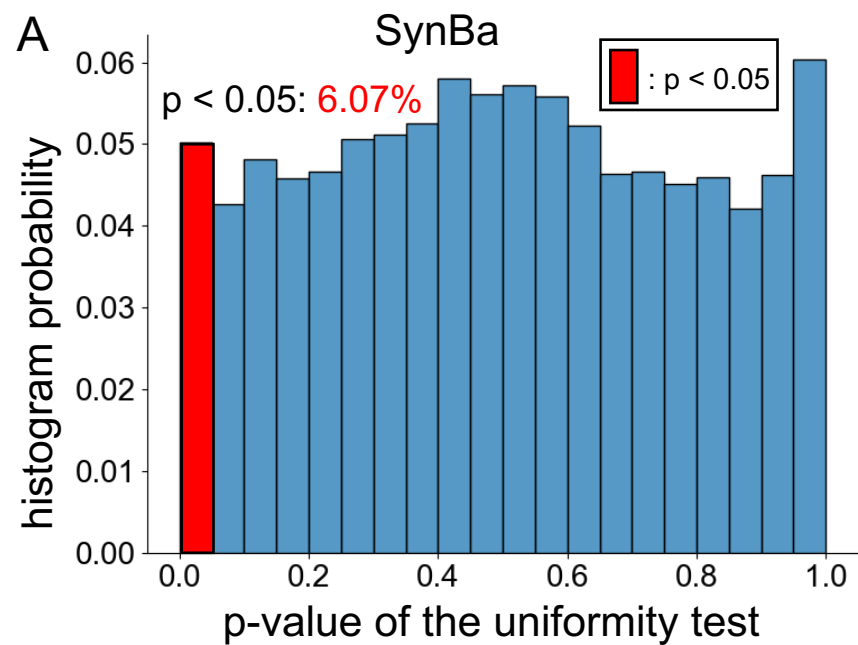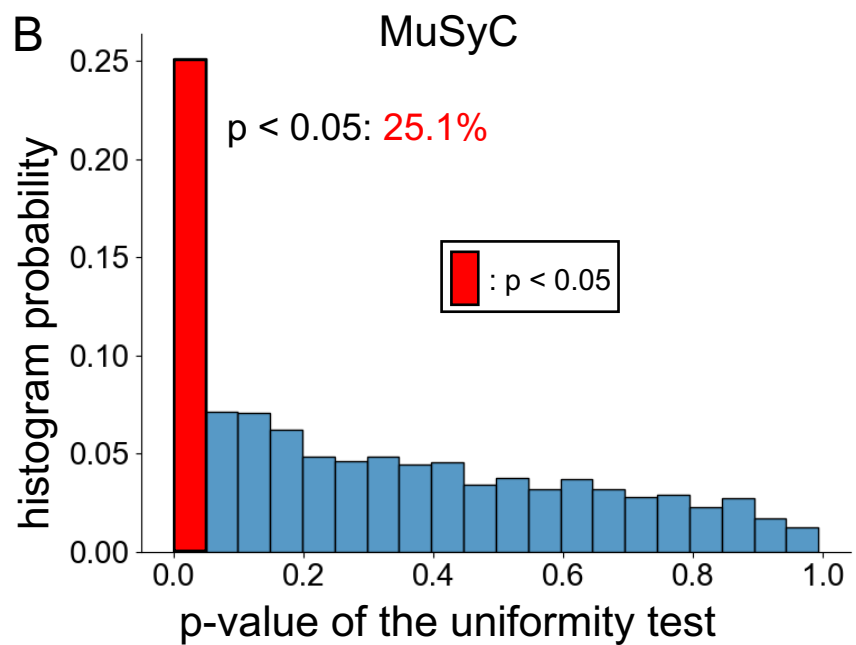

Supplement: btad240_Supplementary_Data [file btad240_supplementary_data.zip › btad240_Supplementary_Data/Zhang.229.fig.S1.pdf]
